# Electron-Beam Inactivation of Human Rotavirus (HRV) for the Production of Neutralizing Egg Yolk Antibodies

**DOI:** 10.3389/fimmu.2022.840077

**Published:** 2022-03-14

**Authors:** Jill W. Skrobarczyk, Cameron L. Martin, Sohini S. Bhatia, Suresh D. Pillai, Luc R. Berghman

**Affiliations:** ^1^ Department of Poultry Science, Texas A&M University, College Station, TX, United States; ^2^ National Center for Electron Beam Research, Texas A&M University, College Station, TX, United States; ^3^ Department of Food Science and Technology, Texas A&M University, College Station, TX, United States; ^4^ Department of Veterinary Pathobiology, Texas A&M University, College Station, TX, United States

**Keywords:** egg yolk, neutralizing, antibody, rotavirus, electron beam, IgY, chicken

## Abstract

Electron beam (eBeam) inactivation of pathogens is a commercially proven technology in multiple industries. While commonly used in a variety of decontamination processes, this technology can be considered relatively new to the pharmaceutical industry. Rotavirus is the leading cause of severe gastroenteritis among infants, children, and at-risk adults. Infections are more severe in developing countries where access to health care, clean food, and water is limited. Passive immunization using orally administered egg yolk antibodies (chicken IgY) is proven for prophylaxis and therapy of viral diarrhea, owing to the stability of avian IgY in the harsh gut environment. Since preservation of viral antigenicity is critical for successful antibody production, the aim of this study was to demonstrate the effective use of electron beam irradiation as a method of pathogen inactivation to produce rotavirus-specific neutralizing egg yolk antibodies. White leghorn hens were immunized with the eBeam-inactivated viruses every 2 weeks until serum antibody titers peaked. The relative antigenicity of eBeam-inactivated Wa G1P[8] human rotavirus (HRV) was compared to live virus, thermally, and chemically inactivated virus preparations. Using a sandwich ELISA (with antibodies against recombinant VP8 for capture and detection of HRV), the live virus was as expected, most immunoreactive. The eBeam-inactivated HRV’s antigenicity was better preserved when compared to thermally and chemically inactivated viruses. Additionally, both egg yolk antibodies and serum-derived IgY were effective at neutralizing HRV *in vitro*. Electron beam inactivation is a suitable method for the inactivation of HRV and other enteric viruses for use in both passive and active immunization strategies.

## Introduction

Routine pathogen inactivation methods in the pharmaceutical industry include chemical and thermal treatments. Both are lengthy procedures, and the former requires additional processing to remove chemical residue from the final formulation. Chemical and heat inactivation also pose a greater risk of protein denaturation, thus compromising antigenicity typically defined as the capacity to be recognized by an antigen-specific antibody. Electron beam (eBeam) inactivation of pathogens is a commercially proven method for the inactivation of microorganisms used extensively in the medical device sterilization and food processing industries (1–4). In this technology, highly energetic electrons are accelerated to 99.999% the speed of light, in “accelerators” and guided into a single beam. Microbial inactivation results from both direct damage to the nucleic acids by electrons or indirectly from high reactive radiolytic species produced by the radiolysis of water molecules by the energetic electrons. Both single and double strand breaks can be generated rendering the organism inactive. Studies in our laboratories have shown that bacterial cells when inactivated retain their metabolism for specific periods of time in a state termed, Metabolically Active yet Non-culturable (MAyNC) (4). This technology is used commercially for different applications (2, 3), but its use for pathogen inactivation in pharmaceutical development is relatively new. High energy eBeam (HEEB) equipment are bulky and require large concrete structures and, therefore, often difficult to incorporate into existing production lines. However, major advances in low energy eBeam (LEEB) technology have resulted in equipment that require minimal shielding and with relatively small equipment footprint (3). The availability of LEEB technology now facilitates the adoption of eBeam technology as in-line equipment for the microbial target inactivation in pharmaceutical production (2). Previous studies have compared electron beam irradiation to traditional methods of pathogen inactivation and concluded that eBeam is an effective alternative (2, 3, 5).

Rotavirus is the leading cause of severe gastroenteritis in infants, children, and at-risk adults (6–10). Infection results in severe diarrhea, dehydration, and in some cases, death. With over 111 million cases, 2 million hospitalizations, and 300,000 deaths each year, rotavirus infections are a global issue (11). Vaccination is the primary method of rotaviral diarrhea prevention. RotaTeq™ and Rotarix™ are pentavalent and monovalent, live-attenuated rotavirus commercial vaccines, respectively. They both induce cross-protective antibody responses against multiple virus strains. While these two commercial vaccines are effective in the United States (88% and 90%, respectively), they’ve had minimal success in developing countries (12). As a result, developing countries have experienced over 80% of all rotavirus-related deaths (11). The poor access to healthcare and costs associated with vaccine storage, transport, and refrigeration make it difficult for these countries to provide adequate protection through vaccination (12, 13). Additional prophylactic strategies should be developed to combat the infection burden in developing countries.

Chickens accumulate antibodies in the egg yolk as a source of maternal immunity for the developing chick (14–16). Egg yolk antibodies (IgY) have a proven record of protecting against viral infections by passive immunity that blocks the adhesion to and invasion of the intestinal mucosa. This is because egg yolk antibodies are more resistant to some of the harsh gut conditions where these infections occur (14, 16). IgY is stable at pH 4-9 and temperatures up to 65°C. Administration as a lyophilized egg yolk powder may further stabilize the antibodies at even lower pH and higher temperatures (17). Additionally, the absence of a hinge region linking the Fab and Fc fragments minimizes cleavage by endogenous proteases (17). Studies have shown that anti-rotavirus egg yolk antibodies are therapeutically effective and significantly reduce the duration of diarrhea in a variety of hosts (16, 18–21). The objective of this study was to explore the utility of eBeam technology for inactivating human rotavirus and generating neutralizing egg yolk antibodies.

## Materials and Methods

The protocols and approvals by the Texas A&M Institutional Animal Care and Use Committee (IACUC) and the Texas A&M Office of Biosafety served as a guide for all animal and microbiota studies. Animal Use Protocol (AUP) #2018-0127 and Institutional Biosafety Committee (IBC) permit #2019-005 provided support for all animal immunization and rotavirus studies, respectively. Hens were housed at the Texas A&M Poultry Science Research Center and eBeam inactivation was performed at the university’s National Center for Electron Beam Research (NCEBR) per biosafety protocols.

### Virus and Cells

Tissue culture adapted HRV Wa (G1 P[8]) and fetal monkey kidney derived host cells, MA104, were purchased from ATCC. Cells were maintained in complete Dulbecco’s Essential Medium containing 8% Fetal Bovine Serum (Atlanta Biologicals), 1% Glutamax, and 1% antibiotic-antimycotic (10,000 units/mL of penicillin, 10,000 µg/mL of streptomycin, and 25 µg/mL of amphotericin B; Gibco). The virus was activated with trypsin (Gibco) at a concentration of 15μg/ml at 37°C for 1 hour. Next, the activated virus was adsorbed onto monolayers of confluent MA104 cells at 37°C for 90 minutes. The cells were re-fed with serum-free Dulbecco’s Modified Essential Medium (Corning) containing 1.5μg/ml trypsin, 1% antibiotic-antimycotic (10,000 units/mL of penicillin, 10,000 µg/mL of streptomycin, and 25 µg/mL of amphotericin B; Gibco), and 25mM HEPES (Gibco), and incubated at 37°C for 7 days or until cytopathic effect (CPE) was observed (22, 23). Virus stocks were titered using a 50% Tissue Culture Infectious Dose (TCID50) assay as described previously (24). The highest dilution of virus that produced CPE in 50% of the infected cells was considered as the endpoint. The titer of the virus was calculated using the Karber method and expressed as log_10_ TCID50/ml (25).

### Electron Beam Inactivation of HRV

The eBeam inactivation was performed at the eBeam facility of NCEBR at Texas A&M University. A 10-MeV, 15-kW linear accelerator delivered the eBeam dose. An initial dose-response (D-10) study was performed to determine the minimum eBeam dose to achieve a 1-log or 90% reduction in virus titer. Determining the D-10 value is critical when attempting to inactivate large microbial titer preparations. Ensuring that uniform eBeam doses are applied in D-10 studies, three replicates of 5ml of virus were triple-packaged in Whirl-pac bags and sealed for each dose point. An extensive set of preliminary studies was performed to ensure that the bags containing viral cultures could be irradiated effectively with dose-uniformity ratio (DUR) as close to 1.0 as possible. The DUR is the most important criterion in irradiation experiments to ensure dose uniformity within samples. Alanine dosimeters were placed at strategic positions on the sample bags to verify the delivered dose. Dosimetry was performed using validated alanine standards that were traceable to international standards (1, 5). The target doses for virus inactivation in the D-10 study were 0 kGy (non-irradiated control), 2.0 kGy, 5.0 kGy, 10.0 kGy, and 15.0 kGy. The measured doses (as determined by alanine dosimetry standards) were 1.99 kGy, 5.1 kGy, 10.15 kGy, and 15.0 kGy. After irradiation, the virus titers were quantified by TCID50 assay. All treatments were repeated at least 3 times. Based on the D-10 value, the minimum eBeam dose required to achieve complete inactivation in 200 mL of high titer rotavirus preparation for immunization studies was determined. All irradiated virus was subject to titration by TCID50 to confirm complete inactivation.

### Chemical and Thermal Inactivation of HRV

Chemical inactivation consisted of incubation of the rotavirus-containing cell culture supernatants at 25°C for 30 minutes with a final concentration of 2% (v/v) formaldehyde followed by 37°C for 30 minutes (26). For thermal inactivation, rotavirus preparations were diluted 1:10 in HBSS prior to incubation at 60°C for 2 hours. After each procedure, the viruses were dialyzed against three changes of PBS for 4 hours at 4°C. The concentrations of both inactivated virus samples were normalized by UV absorbance at 280nm and stored at -80°C.

### Production of Chicken Anti-VP8 Antibodies

Five, 18-week -old, single comb, White Leghorn hens housed at the Texas A&M Poultry Science Center, College Station, TX were used to produce HRV viral protein 8 (VP8)-specific antibodies for ELISAs. Immunizations consisted of 50 µg of recombinant VP8 (Bon-Opus) suspended in 0.3 ml PBS, pH 7.4, mixed with Montanide ISA 71 R VG adjuvant (Seppic) in a 3:7 ratio. All immunizations were administered subcutaneously (s.c.) in the wing-web. Birds were boosted every 2 weeks and VP8-specific antibody titers were monitored by ELISA. Once birds were hyperimmunized as determined by indirect ELISA, 5ml of blood was drawn from each bird, pooled and centrifuged (3,000 x g) to collect the serum. The antibodies were precipitated from the pooled serum using 20% (w/v) powdered ammonium sulfate (27). The pellet containing the enriched antibody was dissolved in PBS, pH 7.4 and quantified by absorbance at 280 nm. Half of the antibody was biotinylated using the EZ Link Sulfo-NHS-Biotin kit (ThermoFisher) before storage at -20°C.

### Characterization of Chicken Anti-VP8 Antibody

The affinity of the anti-VP8 antibody was determined by indirect ELISA and Western blot. For the ELISA, a flat-bottom ELISA plate (Corning) was coated overnight with 5µg/ml purified HRV VP8. The plate was blocked with 2% BSA in PBS. The anti-VP8 antibody was added to the plate (1:1,000) and incubated for 1 hour at 37°C. After rinsing the plate with PBST, HRP-conjugated goat anti-chicken IgY (1:3,000) was added and incubated for 1 hour at room temperature (Jackson ImmunoResearch). TMB substrate (SeraCare) was used as the enzyme substrate and the plate was read at 450nm. For the Western blot, 5µg of purified recombinant VP8 protein was separated by SDS-PAGE using a 12% Tris-Glycine gel (Invitrogen) and transferred to a PVDF membrane using the TransBlot Turbo Transfer System (BioRad). The membrane was blocked in 2% BSA overnight at 4°C. The purified, biotinylated chicken anti-VP8 antibody served as the primary antibody to confirm immunoreactivity with recombinant VP8 protein. The membrane was incubated with anti-VP8 for 1 hour at room temperature (1:3,000) followed by incubation with streptavidin-conjugated HRP (1:10,000). The membrane was developed with a chemiluminescent substrate solution (Agilent) and imaged on a ChemiDoc (BioRad).

### Virus Antigenicity ELISA

The antigenicity of HRV before and after eBeam inactivation was assessed by a sandwich enzyme-linked immunosorbent assay. Briefly, a flat-bottom ELISA plate (Corning) was coated overnight with 5µg/ml purified chicken anti-HRV VP8 as the capture antibody. The use of an antibody raised against one of the outer capsid proteins allowed for the comparison of antibody recognition of an important neutralizing protein. The ability of the purified chicken anti-VP8 antibody to bind to a virus after inactivation indicated that its antigenic integrity was maintained. The plate was blocked with 5% gelatin in PBS. A single batch of four HRV stocks, including live virus, electron beam-inactivated virus, chemically inactivated virus, and thermally inactivated virus, was serially diluted 2-fold starting with a 400µg/ml concentration. The diluted virus preparations were added to the plate and incubated for 1 hour at 37°C. Biotinylated chicken anti-HRV VP8 was added as the detection antibody (1:1,000) and incubated for 1 hour at 37°C. HRP-conjugated neutravidin was added as the enzyme (1:20,000) (ThermoFisher) and TMB substrate (SeraCare) was used to develop the color. Sulfuric acid (50 µl of a 2 M solution) was added to stop the enzymatic reaction and the plate was read at 450nm in a BioTek microplate reader (Synergy H1).

### Purification of Virus

The eBeam inactivated HRV was purified by ultracentrifugation using a cesium chloride gradient as described previously (23). After the final spin, the top fraction containing triple-layered virus particles was isolated and dialyzed against sterile PBS, pH 7.4 overnight. The concentration of purified virus particles was normalized by UV absorbance at 280nm and stored at -80°C.

### Immunization of Hens and Production of Egg Yolk Antibodies

Five, 18-week-old, single comb, White Leghorn hens housed at the Texas A&M Poultry Science Center, College Station, TX were used to produce HRV-specific antibodies. Immunizations consisted of 50 µg of HRV suspended in 0.3 ml PBS, pH 7.4, mixed with Montanide ISA adjuvant (Seppic) in a 3:7 ratio. All immunizations were administered subcutaneously (s.c.) in the wing-web. Birds were boosted every 2 weeks and blood samples were collected after each boost to quantify HRV-specific antibody levels by ELISA. Four immunizations were administered before the birds were hyperimmunized as determined by indirect ELISA. Eggs were then collected daily for 1 week, and frozen at -20°C until needed. Pre-immune eggs were also collected from each hen before receiving any immunizations.

### Purification of IgY From Egg Yolks

Frozen eggs were thawed in room-temperature DI water and yolks were separated from the egg white. To remove the lipids, yolks were pooled and emulsified in DI water before adjusting the pH to 7.0 and freezing again at -20°C. The yolk solution was thawed again the next day and total IgY was precipitated using 20% (w/v) powdered ammonium sulfate (27). The pellet containing highly enriched IgY was dissolved in PBS, pH 7.4, dialyzed against additional PBS, quantified by absorbance at 280 nm and stored at -20°C.

### Detection of Anti-HRV Antibodies in Serum and Egg Yolk

The presence of anti-HRV IgY antibodies in serum and egg yolk from hyperimmunized hens was monitored and assayed by a modified indirect ELISA as described previously (28). Briefly, 10 µg/ml of purified HRV was coated on microtiter plates (Corning) overnight and allowed to react with serum and yolk samples diluted 1:1000. In the titration ELISA, the serum and yolk samples were serially diluted three- or five-fold. Antibodies were diluted in PBS, pH 7.4 containing 2% (w/v) BSA. Upon rinsing 3X with 200µl PBST, specifically bound anti-HRV IgY was probed by horseradish peroxidase-conjugated goat anti-chicken IgY (Jackson Immunolabs) at 1:3,000 dilution, followed by detection with Tetramethylbenzidine High Kinetics substrate (BioVision). A positive ELISA value was identified as >2X mean OD450 value of pre-immune IgY (at 1:1,000).

### *In Vitro* Virus Neutralization Assay

The virus neutralization titer of anti-HRV IgY was determined using MA104 cell monolayers by methods described previously (23, 29). The crude serum and egg yolk preparations contained 20 and 5mg/ml of antibody, respectively. The serum and yolk samples were normalized to a concentration of 5mg/ml before diluting 25 microliters of both antibody preparations in 25 microliters of media to create a 2-fold serial dilution for the assay. Two hundred TCID50 units of HRV were incubated with the 2-fold serial dilution of IgY at 37°C for 1 hour. The antibody-virus suspension was layered onto confluent MA104 cell monolayers grown in 96-well microplates (Nunc, USA). After 7 days of incubation at 37°C in a humidified 5% CO_2_ atmosphere, the plates were examined for the presence of CPE. The complete absence of CPE was scored as positive for neutralization. Antibody samples were run in triplicate and the reciprocal of the mean highest dilution of IgY was reported as the neutralization titer.

## Results

### Complete Inactivation of HRV Was Achieved at 15 kGy

The titer of the propagated HRV stock was quantified by TCID50 and reported as 6.5 log TCID50. This titer was maintained for all inactivation and immunization experiments. The propagated virus was purified by ultracentrifugation with a cesium chloride gradient. Purification of HRV yielded two distinct bands in the density gradient corresponding to double and triple layered virus particles. The double and triple layered particles settled at a density of 1.37 g/cm^3^ and 1.34 g/cm^3^, respectively.

**Figure 1:**
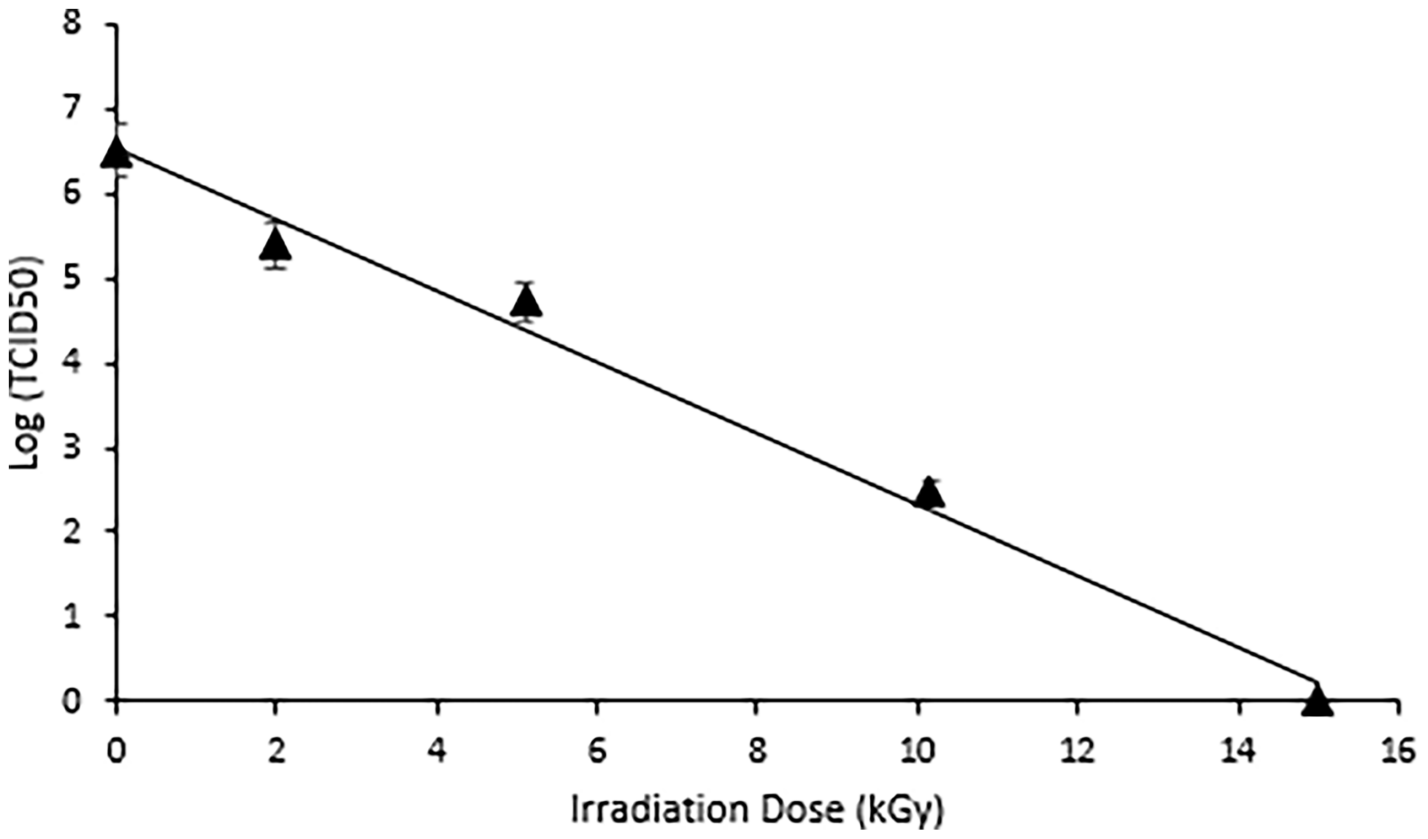
Complete Inactivation of HRV was achieved at 15 kGy. Dose response curve illustrating the effects of electron beam irradiation on HRV stocks. The target doses were set at 0, 2, 5, 10, and 15 kGy and the measured doses were 1.99 kGy, 5.1 kGy, 10.15 kGy, and 15.0 kGy. A TCID50 assay was used to quantify the reduction in viral titer. The D_10_ value, or dose required to achieve a 1-log reduction in viral titer was calculated as 2.38 (± 0.017) kGy. Complete inactivation was achieved at 15kGy.

While viruses are more resistant than bacteria to ionizing radiation (because of the smaller genome size), they are, nevertheless, still susceptible to eBeam doses. The dose (± standard error [SE]) required to achieve a 1-log or 90% reduction (D-10 value) in HRV titer was calculated at 2.38 (± 0.017) kGy, while total inactivation of the virus was achieved at 15 kGy (**Figure 1**). The absence of CPE observed after irradiation at 15 kGy indicated no viral replication had occurred and the virus was deemed non-infectious. This eBeam dose was used in all subsequent experiments for complete inactivation of HRV stocks.

### Anti-VP8 Chicken Antiserum Detects Recombinant VP8 in ELISA and Immunoblot

**Figure 2:**
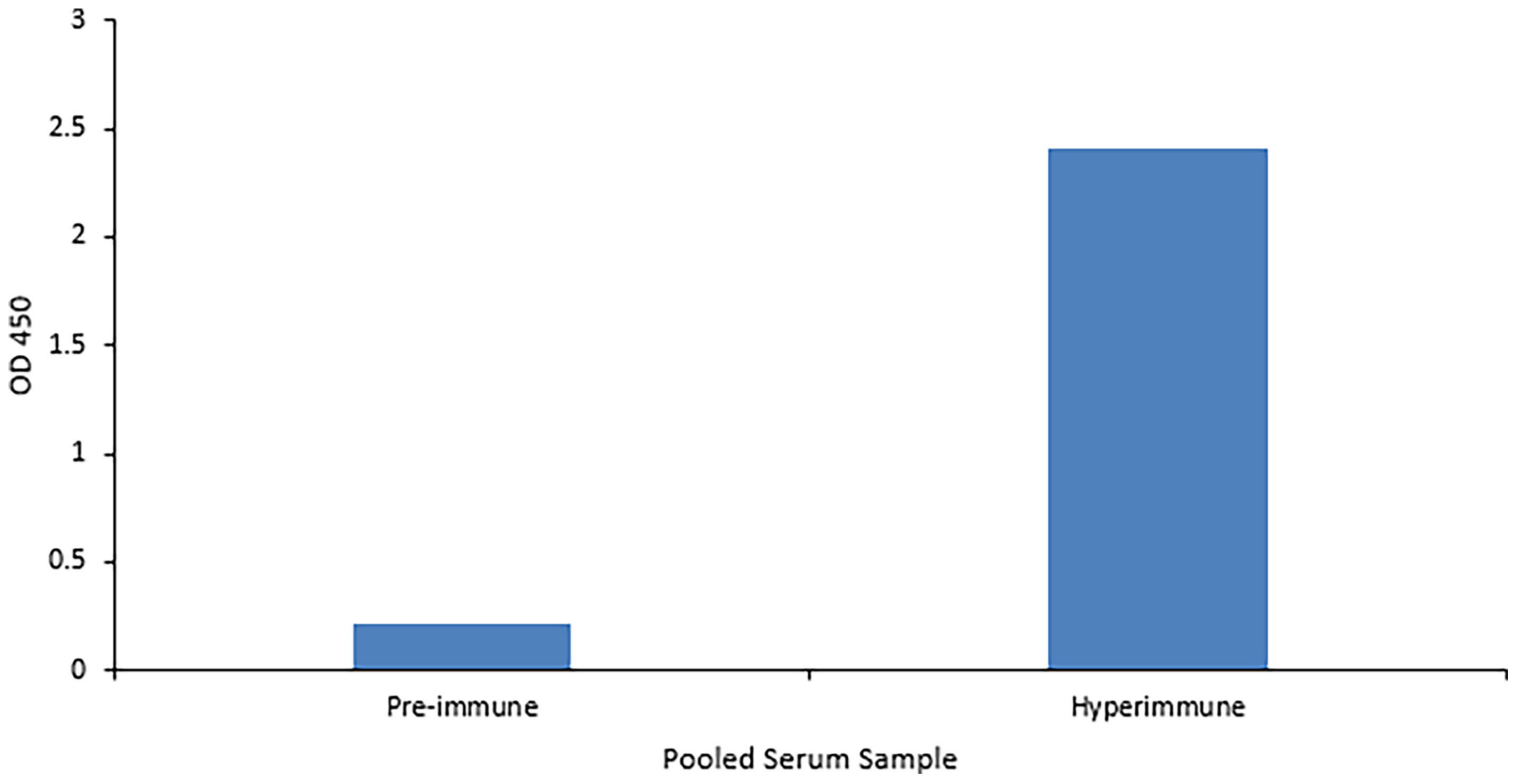
Anti-VP8 chicken antiserum detects recombinant VP8 in an ELISA. Indirect ELISA comparing the affinity of pooled pre- and hyper-immune serum antibodies for the recombinant VP8 antigen. Birds received three immunizations consisting of 50µg purified VP8 with Montanide as an adjuvant. An ELISA plate was coated overnight with 5µg/ml purified HRV VP8 and blocked with 2% BSA in PBS. The pre- and hyper-immune anti-VP8 serum antibodies served as the primary antibody (1:1,000). HRP-conjugated goat anti-chicken IgY was added as the secondary antibody (1:3,000). The hyperimmune serum was specific for the viral protein.

**Figure 3:**
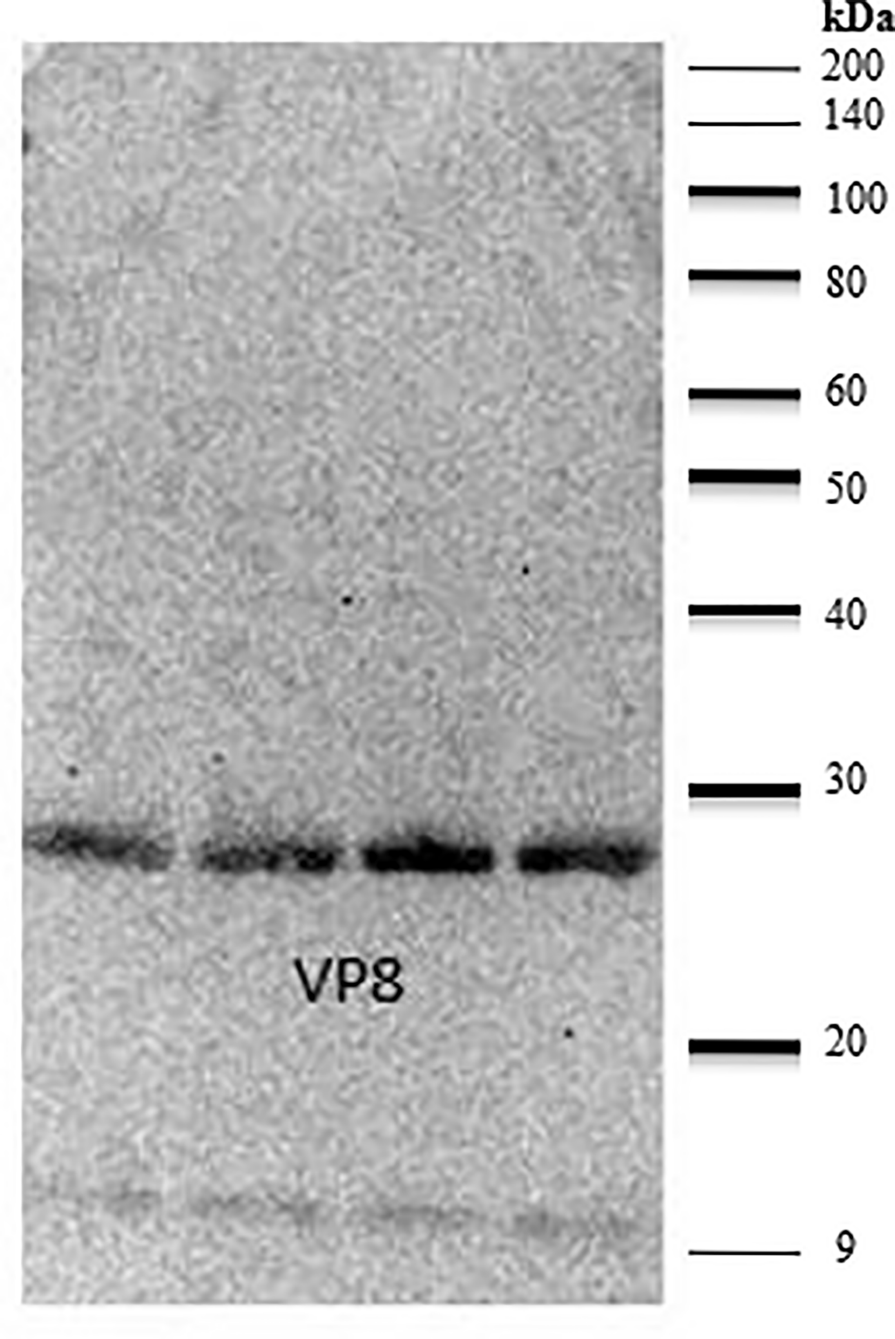
Anti-VP8 chicken antiserum detects recombinant VP8 in a Western blot. Lanes 1-4 were loaded with 5µg of purified recombinant VP8, separated by SDS-PAGE and transferred to a PVDF membrane. The membrane was blocked in 2% BSA. Purified, biotinylated chicken anti-VP8 antibody served as the primary antibody (1:3,000) and streptavidin conjugated HRP was used to detect (1:10,000). Lanes 1-4 were loaded with 5µg of purified recombinant VP8. The purified, biotinylated chicken anti-VP8 antibody recognized the 28 kDa protein at a dilution of 1:3,000.

The ability of the purified chicken anti-VP8 antibody to bind to recombinant and native VP8 was demonstrated with two assays. The signal of the hyperimmune antiserum was 11 times that of the pre-immune serum indicating that the birds were hyperimmunized and the serum antibodies were specific for the recombinant viral protein (**Figure 2**). In addition, immunoblotting analysis of the recombinant VP8 protein using chicken anti-VP8 produced a single band at the expected apparent molecular weight of 28 kDa (**Figure 3**).

### Electron Beam Inactivation Was Effective at Preserving Antigenicity of HRV

**Figure 4:**
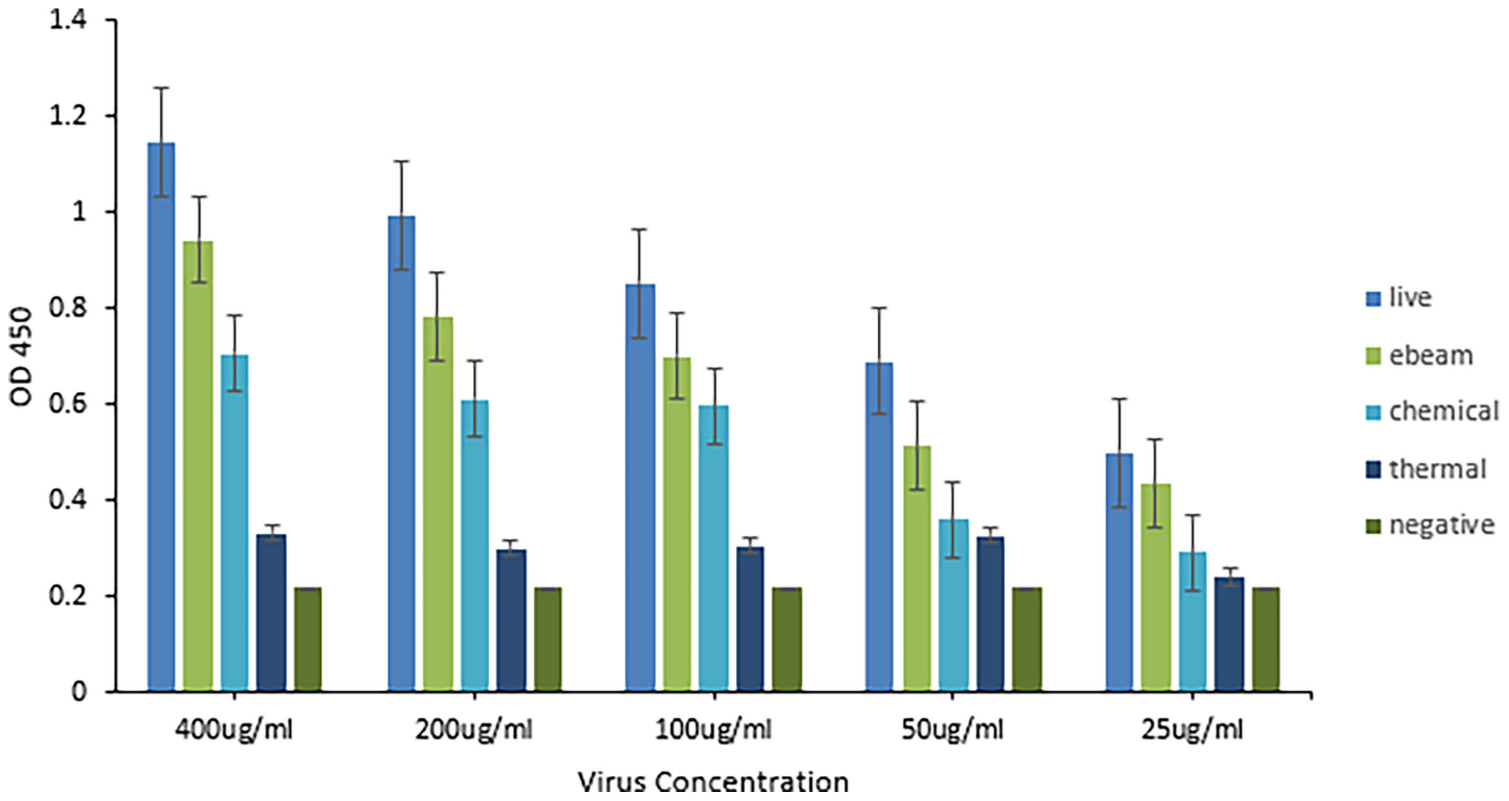
Electron beam inactivation was superior in preserving antigenicity of HRV. Sandwich ELISA titration of inactivated HRV stocks to determine antigenicity. An ELISA plate was coated with purified chicken anti-HRV VP8 as the capture antibody and blocked with 5% gelatin in PBS. Four different HRV stocks including live virus, electron beam-inactivated virus, chemically inactivated virus, and thermally inactivated virus, were serially diluted starting with a 400µg/ml concentration. Biotinylated chicken anti-HRV VP8 served as the detection antibody (1:1,000) and HRP-conjugated neutravidin was used to detect (1:20,000). The eBeam inactivated virus was detected at ~75-90% that of non-irradiated live virus control. The chemical and thermal inactivated viruses were detected at ~50-60 and 25-40% that of the live virus, respectively. Compared to the live virus, antigenicity was best maintained after eBeam inactivation.

A sandwich ELISA using anti-HRV VP8 antibodies was performed to compare the antigenicity of the virus before and after the three different inactivation methods (**Figure 4**). A linear relationship was observed between virus concentration and measured OD450. The live (non-inactivated) control consistently reported the highest OD450 followed by eBeam inactivated, chemically, and thermally inactivated virus preparations. After eBeam inactivation at 15 kGy, the virus was detected at ~75-90% that of non-irradiated live virus control. The chemical and thermal inactivated viruses were detected at ~50-60 and 25-40% that of the live virus, respectively. A signal to noise ratio (SNR) greater than 2 was reported for only the live and eBeam inactivated viruses at 25 µg/ml. When compared to traditional chemical and thermal inactivation methods, eBeam inactivation exhibited improved conservation of antigenicity and chemical and thermal methods of inactivation were more detrimental to the virus’ antigenic integrity.

### Chickens Reached Maximal Anti-HRV Titers After Three Injections

**Figure 5:**
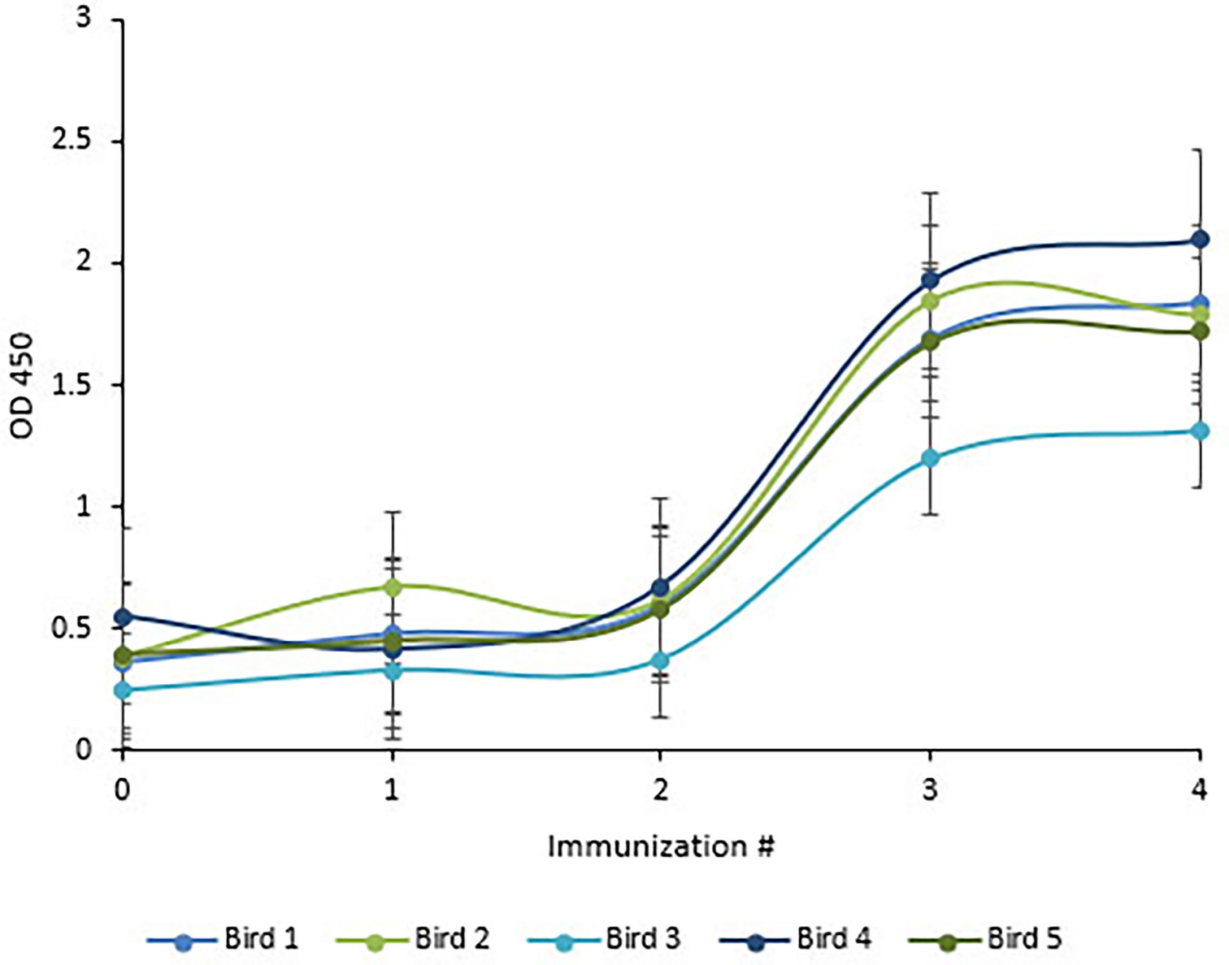
Chickens reached maximal anti-HRV titers after three immunizations. ELISA quantification of chicken anti-HRV specific serum antibody level in response to immunization. An ELISA plate was coated with 10µg/ml of purified HRV and blocked with 2% BSA in PBS. Pre- and hyper-immune serum antibodies served as the primary antibody (1:1000). HRP-conjugated goat anti-chicken IgY served as the secondary antibody (1:3,000). Titers peaked after the third immunization and birds were considered to be hyperimmunized.

**Figure 6:**
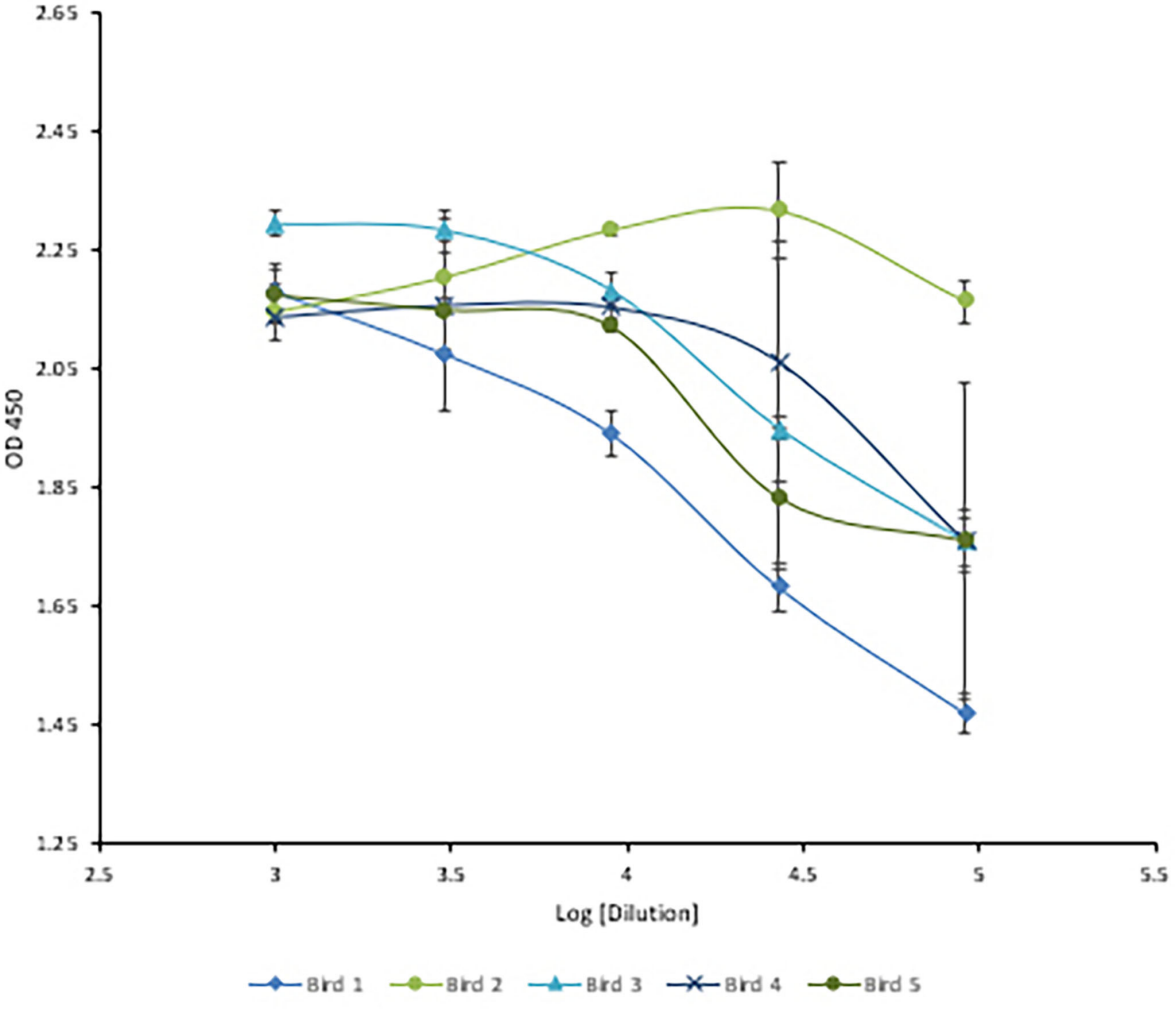
Three-fold serial titration of anti-HRV titers after three immunizations. Titration of hyperimmune anti-HRV serum using a 3-fold serial dilution. An ELISA plate was coated with 10µg/ml of purified HRV and blocked with 2% BSA in PBS. Serum and yolk antibodies were serially diluted three-fold in PBS containing 2% BSA. HRP-conjugated goat anti-chicken IgY served as the secondary antibody (1:3,000). At 1/91,000 [log (4.95)] the signal from bird #2 was still comparable to the undiluted serum.

**Figure 7:**
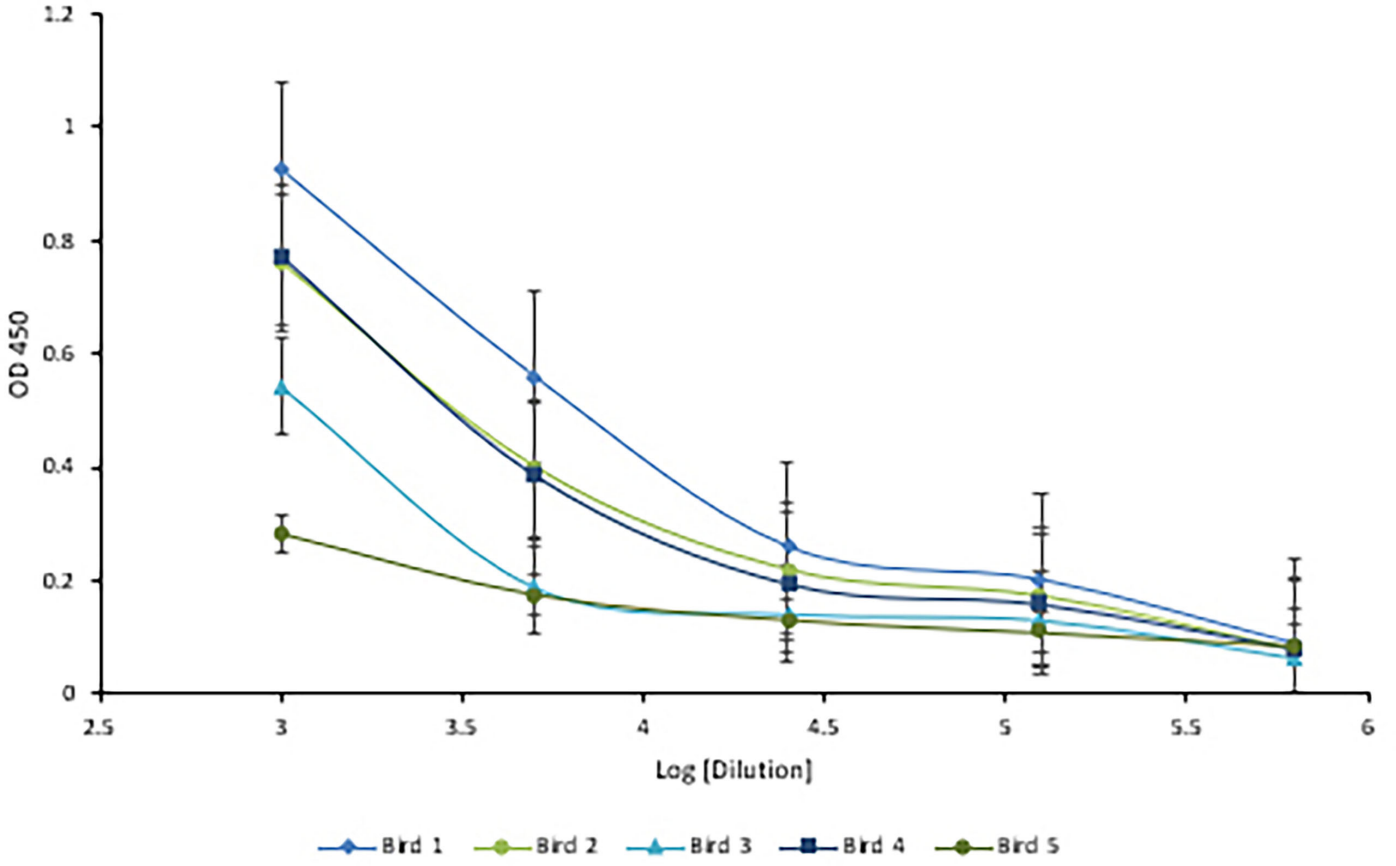
Five-fold serial titration of anti-HRV titers after three immunizations. Titration of hyperimmune anti-HRV serum using a 5-fold serial dilution. An ELISA plate was coated with 10µg/ml of purified HRV and blocked with 2% BSA in PBS. Serum and yolk antibodies were serially diluted five-fold in PBS containing 2% BSA. HRP-conjugated goat anti-chicken IgY served as the secondary antibody (1:3,000). A dilution of 125,000 (log [5.09]) was identified as the detection limit.

Following immunization, the hens’ HRV specific antibody level was monitored by indirect ELISA. Anti-HRV IgY titers peaked after the third immunization and were maintained thereafter (**Figure 5**). As expected, the response was highly variable between biological replicates. (ELISA S/N values: 4.34, 2.15, 2.55, 3.24, and 1.46). Once the HRV-specific antibody titer plateaued, birds were considered hyperimmunized. The maximum titer, defined as the reciprocal of the highest dilution producing a statistically significant indirect ELISA signal was 125,000 (**Figures 6**, **7**).

### Serum- and Egg Yolk-Derived IgY Demonstrate *In Vitro* Virus Neutralization Activity

Virus neutralization was defined as the complete absence of CPE. Neutralization titers were reported as the reciprocal of the highest antibody dilution capable of preventing infection or CPE development. Serum neutralization titers were approx. 2-fold higher than corresponding egg yolk titers, ranging from 863-1706 in the serum and 341-768 in the egg yolk (**Table 1**). Pre-immune serum and egg yolk IgY were negative for neutralization.

**Table 1:** *In Vitro* Virus Neutralization Titers.

Bird #	Serum	Egg Yolk
1	1706	341
2	1365	512
3	863	424
4	1365	768
5	1024	424

Virus neutralization titers, defined as the reciprocal of the highest antibody dilution capable of preventing in vitro infection or CPE development.

## Discussion

This study outlines the use of eBeam inactivation to produce high-titer neutralizing HRV-specific egg yolk antibodies and analyzes their potential to prevent infection *in vitro*. Hens were immunized with eBeam inactivated HRV particles from the most common human strain, the Wa (G1P[8]) strain. To inactivate the rotavirus, electrons generated from HEEB equipment were utilized to create extensive breaks in the RNA genome. In the D-10 study, the HRV titer decreased as the dose of electron beam radiation increased, demonstrating an inverse but linear relationship. The dose required to inactivate 1-log of virus was reported as 2.38 + (0.017) which was, as expected, significantly higher than the reported D-10 values for bacterial pathogens. This is generally attributed to the smaller viral genome sizes compared to bacteria (2). After exposure to 15 kGy eBeam dose, high titers of HRV exhibited no replication suggesting the efficacy of the technology. Not only was the virus effectively inactivated but it was done so in a fraction of the time that traditional chemical and thermal inactivation methods require. Some chemical processes can take days or even weeks to fully inactivate. Additionally, the viral antigenicity obtained by dose-optimized electron beam inactivation (~80%) markedly exceeded antigenicity after traditional chemical (~60%) and thermal (~40%) treatments. Nucleic acids are the primary target of ionizing radiation based on the larger G-values compared to proteins or lipids (2). The G-value is defined as the amount of radiolytic species produced per 100 eV of absorbed energy. The eBeam-inactivated rotavirus was recognized at levels closest to that of the non-inactivated, live virus control (**Figure 4**) and, when used for antibody generation, produced impressive HRV-specific antibody titers (**Figures 6**, **7**). These data suggest that eBeam inactivation preserves the key epitopes found on the surface of viral proteins and, therefore, retains optimal immunoreactivity (2). The efficacy of eBeam as an inactivation technology for pharmaceutical development observed in this study is in line with previous reports on influenza A, PRRSV, and the gram-negative bacteria *R. pneumotropicus* confirming the notion that eBeam inactivation of pathogens is less damaging (2, 3, 5).

Not only did the eBeam-inactivated virus maintain antigenicity and generate a high titer of antigen specific antibodies, but these antibodies also demonstrated effective and efficient virus neutralization activity *in vitro*. *In vitro* studies are key to determining drug efficacy in a controlled environment before introduction to a live animal host. These studies allow multiple drugs to be tested at one time and only those that are most effective proceed to *in vivo* studies (30). The serum neutralization titers we observed in this study were approx. 2-fold higher than corresponding egg yolk titers, ranging from 863-1706 in the serum and 341-768 in the egg yolk, which may be explained by the fact that egg yolk IgY needs to be delipidated, precipitated and dialyzed before it is suitable for addition to eukaryotic cell cultures. Egg yolk IgY is commonly purified by ammonium sulfate precipitation, yielding high levels of antibody at greater than 60-70% purity due to the lack of available affinity purification methods for IgY (27, 31). Although the neutralization titer of the hyperimmune egg yolk antibodies in the present study was lower than that of serum, all neutralization titers were comparable to those reported in the literature (14, 16, 18–21). Egg yolk antibodies represent the most prolific and cost-effective polyclonal antibody platform. Chickens are arguably the most inexpensive animal to house and feed. Depending on the age and breed of chicken and antigen used, up to 80mg of total IgY can be precipitated from one egg. Leghorns can produce over 300 eggs in a year (14, 17, 32). In this study, approximately 60 mg of total IgY was harvested from each egg providing 0.6-6 mg of HRV-specific antibody. It has been reported that 1-10% of egg yolk antibody preparations are antigen specific (16). When combined with eBeam inactivation of viruses, egg yolk antibodies provide a unique tool for passive immunization and an alternative prophylactic strategy to combat rotavirus infections. These data have important implications for the use of electron beam irradiation as an effective method of pathogen inactivation in pharmaceutical development.

## Data Availability Statement

The original contributions presented in the study are included in the article/supplementary material. Further inquiries can be directed to the corresponding author.

## Ethics Statement

The animal study was reviewed and approved by Texas A&M Institutional Animal Care and Use Committee (IACUC).

## Author Contributions

JS performed all experiments, analyzed data, and drafted the manuscript. CM assisted with project design and provided experimental advice. SB provided experimental advice. SP conceived the idea of the study and assisted with project design and data analysis. LB also conceived the idea of the study and assisted with project design, support, data analysis and manuscript writing. All authors read and approved the manuscript.

## Funding

This study was funded with internal money from both the SP and LB labs.

## Conflict of Interest

The authors declare that the research was conducted in the absence of any commercial or financial relationships that could be construed as a potential conflict of interest.

## Publisher’s Note

All claims expressed in this article are solely those of the authors and do not necessarily represent those of their affiliated organizations, or those of the publisher, the editors and the reviewers. Any product that may be evaluated in this article, or claim that may be made by its manufacturer, is not guaranteed or endorsed by the publisher.
